# Correction: Caregivers’ mental distress and child health during the COVID-19 outbreak in Japan

**DOI:** 10.1371/journal.pone.0250191

**Published:** 2021-04-08

**Authors:** Sayaka Horiuchi, Ryoji Shinohara, Sanae Otawa, Yuka Akiyama, Tadao Ooka, Reiji Kojima, Hiroshi Yokomichi, Kunio Miyake, Zentaro Yamagata

There is an error in the eighth sentence of the Abstract. The correct sentence is: After adjusting for covariates, child health issues increased among caregivers with moderate mental distress (odds ratio 2.21, 95% confidence interval 1.56–3.12) and severe mental distress (odds ratio 3.11, 95% confidence interval 2.21–4.39) compared with caregivers with no mental distress.

There are also errors in the fifth and seventh sentences in the third paragraph of the Results. The correct fifth sentence is: Odds of child health issues were higher among participants with moderate mental distress (OR 2.21, 95% CI 1.56–3.12) and severe mental distress (OR 3.11, 95% CI 2.21–4.39), compared with those with no mental distress. The correct seventh sentence is: There was no interaction between the effects of the caregiver’s mental status and caregiver’s gender (p = 0.765), caregiver’s age (p = 0.114), child’s gender (p = 0.324), child’s age (p = 0.740), and school closure (p = 0.429) on child health condition.
