# Supplementary material for: Caregivers’ mental distress and child health during the COVID-19 outbreak in Japan
Source: PLoS One. 2020 Dec 10;15(12):e0243702. doi: 10.1371/journal.pone.0243702 (PMC7728265; doi:10.1371/journal.pone.0243702)
Supplement: S1 File — (DOCX) [file pone.0243702.s001.docx]

**【WEB調査（2020-04xxx）】**

| **新型コロナウイルスから子どもの心と生活を守るための実態調査** |
| --- |

| **最初に、あなたの性別や年齢などについておうかがいします。** |
| --- |

**【すべての方に】**

ＳＣ１.あなたの性別について教えてください。（１つだけ）

| 1. 女性 2. 男性 |
| --- |

［改ページ］

ＳＣ２.あなたの年齢を教えてください。（１つだけ）

| 1. ２０歳未満 2. ２０～２４歳 3. ２５～２９歳 4. ３０～３４歳 5. ３５～３９歳 6. ４０～４４歳 7. ４５歳以上 |
| --- |

［改ページ］

ＳＣ３.あなたご自身と同居されている、ご自分のお子さまはいらっしゃいますでしょうか。いらっしゃる場合は、お子さまの性別と年齢をお答えください。（いくつでも）

| 1. 同居している子どもはいない　（→調査終了） 2. ２歳以下の男の子 3. ３歳の男の子（４月初めで幼稚園・保育園の年少クラス） 4. ４歳の男の子（４月初めで幼稚園・保育園の年中クラス） 5. ５歳の男の子（４月初めで幼稚園・保育園の年長クラス） 6. ６歳の男の子（４月初めで小学校１年生） 7. ７歳の男の子（４月初めで小学校２年生） 8. ８歳の男の子（４月初めで小学校３年生） 9. ９歳の男の子（４月初めで小学校４年生） 10. １０歳の男の子（４月初めで小学校５年生） 11. １１歳の男の子（４月初めで小学校６年生） 12. １２歳の男の子（４月初めで中学校１年生） 13. １３歳の男の子（４月初めで中学校２年生） 14. １４歳の男の子（４月初めで中学校３年生） 15. １５歳以上の男の子（４月初めで高校生以上） 16. ２歳以下の女の子 17. ３歳の女の子（４月初めで幼稚園・保育園の年少クラス） 18. ４歳の女の子（４月初めで幼稚園・保育園の年中クラス） 19. ５歳の女の子（４月初めで幼稚園・保育園の年長クラス） 20. ６歳の女の子（４月初めで小学校１年生） 21. ７歳の女の子（４月初めで小学校２年生） 22. ８歳の女の子（４月初めで小学校３年生） 23. ９歳の女の子（４月初めで小学校４年生） 24. １０歳の女の子（４月初めで小学校５年生） 25. １１歳の女の子（４月初めで小学校６年生） 26. １２歳の女の子（４月初めで中学校１年生） 27. １３歳の女の子（４月初めで中学校２年生） 28. １４歳の女の子（４月初めで中学校３年生） 29. １５歳以上の女の子（４月初めで高校生以上） |
| --- |

同居チェック：選択肢１と2～29を同時回答→アラート

［改ページ］

【ＳＣ３で3～14､17～28をいずれか回答（3歳～14歳の男の子・女の子が1人でもいる）の方に】

ＳＣ４.以下の文章をよくお読みいただき、本アンケートに協力いただけるか、ご回答ください。

**【アンケートご協力のお願い】**

この調査は、山梨大学大学院附属出生コホート研究センターからの委託を受けて、（株）日本リサーチセンターが実施しています。

新型コロナウイルス対策で、子どもたちは休校や外出禁止で家の中にいることのストレス、友だちと会えないさみしさ、過剰な報道よる恐怖心などのメンタルヘルスが問題となっています。子どもが怖がって外に出たがらなかったり、奇声をあげたりするなどの異常行動も見られています。そのようなお子様の様子を見て保護者の皆様も不安に感じられているようです。また、海外では家庭内暴力（DV）が増加しているとの報道もあり、今後、わが国でも同様のことが起き、DV、虐待が増加するのではないかと危惧されます。

新型コロナウイルス感染予防と同時に子どもの心の対策は喫緊の課題です。

そこで、山梨大学大学院附属出生コホート研究センターでは、このアンケートを通じて、新型コロナウイルス感染症対策における子ども及び保護者の現状を把握して、今後の対策、アドバイスの基礎資料とします。なお、本調査の結果は集計をして、学会等での発表、論文による公表を予定しています。

本アンケートにご協力を賜りますよう、お願い申し上げます。

アンケートの中では、あなたやあなたのお子さんの健康状態などについておたずねいたしますが、お答えいただいた内容は、個人が特定できないようにし、山梨大学大学院附属出生コホート研究センターでの統計的な分析にのみ利用しますので、あなた様のお名前やご回答内容をセールスなど他の目的に利用することは決してございません。

この調査にご協力いただけるか、ご回答ください。（１つだけ）

| 1. このアンケートに協力できる　（→ＳＣ１へ） 2. このアンケートには協力できない　（→調査終了） |
| --- |

［改ページ］

ＳＣ４で「１．このアンケートに協力できる」と回答の方

↓

本調査（問１）へ

| **以下について、それぞれあなたにあてはまるものをご回答ください。** |
| --- |

問１．お住まいの都道府県について教えてください。（１つだけ）

| 1. 北海道 2. 青森県 3. 岩手県 4. 宮城県 5. 秋田県 6. 山形県 7. 福島県 8. 茨城県 9. 栃木県 10. 群馬県 11. 埼玉県 12. 千葉県 13. 東京都 14. 神奈川県 15. 新潟県 16. 富山県 17. 石川県 18. 福井県 19. 山梨県 20. 長野県 21. 岐阜県 22. 静岡県 23. 愛知県 | 1. 三重県 2. 滋賀県 3. 京都府 4. 大阪府 5. 兵庫県 6. 奈良県 7. 和歌山県 8. 鳥取県 9. 島根県 10. 岡山県 11. 広島県 12. 山口県 13. 徳島県 14. 香川県 15. 愛媛県 16. 高知県 17. 福岡県 18. 佐賀県 19. 長崎県 20. 熊本県 21. 大分県 22. 宮崎県 23. 鹿児島県 24. 沖縄県 |
| --- | --- |

［改ページ］

問２．お住まいの市区町村の人口規模について教えてください。（１つだけ）

| 1. ５０万人以上 2. ３０万人～５０万人未満 3. １０万人～３０万人未満 4. ５万人～１０万人未満 5. ３万人～５万人未満 6. １万人～３万人未満 7. １万人未満 |
| --- |

［改ページ］

| **以下は、あなたご自身と、同居されている「●歳の●の子」についておうかがいします。** |
| --- |

　　※「●」はＳＣ３の回答を表示（複数回答している場合は回答の選択肢からランダムで固定表示）
以下も同様です。

問４－１．あなたのお子さま**「●歳の●の子」**の園や学校の休校の状況について教えてください。

　　　　　園や学校は、現在休園・休校されていますか。（１つだけ）

| 1. 現在、休園・休校している 2. 現在、休園・休校していない |
| --- |

［改ページ］

**【問４－１で「１　現在、休園・休校している」と回答の方に】**

問４－２．あなたのお子さま**「●歳の●の子」**の園や学校の休校の状況について教えてください。

　　　　　次のうち、あてはまるものを選んでください。（いくつでも）

| 1. 特別に通園、通学している 2. 学童を利用している 3. オンライン授業を利用できる 4. 上記にあてはまるものはない |
| --- |

同居チェック：選択肢１～3と4を同時回答→アラート

［改ページ］

**【問４－１で「１　現在、休園・休校している」と回答の方に】**

問４－３．あなたのお子さま**「●歳の●の子」**の園や学校の休校開始時期について教えてください。

　　　　　　　　　　　月 　　　　　　　　　　　　日

　　　（２～５の回答制御）　　　　（１～３１の回答制御）

問４－４．あなたのお子さま**「●歳の●の子」**の園や学校の休校終了予定時期について教えてください。

　　　　　　　　　　　月 　　　　　　　　　　　　日　（予定）

　　　（５～６・未定の回答制御）　　　　（１～３１・未定の回答制御）

［改ページ］

問5．あなた及びあなたのパートナー（奥様や旦那様など）の勤務状況を教えてください。

問5－１．あなたの仕事は何ですか。（１つだけ）

| 1. 会社員 2. 公務員 3. 自営業・自由業 4. 農林漁業 5. パート・アルバイト 6. 家事専業（自宅） 7. 学生 8. 無職 9. その他（　　　　　　　　　　　） |
| --- |

［改ページ］

問5－２．あなたは月曜日から金曜日で、日中、お子さま**「●歳の●の子」**と一緒にいる時間はどれくらいですか。　（１つだけ）

| 1. ほぼ終日、一緒にいる 2. 半日程度、一緒にいる 3. 日中はほとんど一緒にいない |
| --- |

［改ページ］

問5－３．あなたのパートナー（奥様や旦那様など）の仕事は何ですか。（１つだけ）

| 1. 会社員 2. 公務員 3. 自営業・自由業 4. 農林漁業 5. パート・アルバイト 6. 家事専業（自宅） 7. 学生 8. 無職 9. その他（　　　　　　　　　　　） |
| --- |

［改ページ］

問5－４．あなたのパートナー（奥様や旦那様など）は月曜日から金曜日で、日中、お子さま**「●歳の●の子」**と一緒にいる時間はどれくらいですか。（１つだけ）

| 1. ほぼ終日、一緒にいる 2. 半日程度、一緒にいる 3. 日中はほとんど一緒にいない |
| --- |

［改ページ］

問６．あなたが、下記についてで困っていることがあればお答えください。（いくつでも）

| 1. 自分の仕事・家事 2. パートナー（奥様や旦那様など）の仕事・家事 3. 子ども**「●歳の●の子」**を保育園や学童等であずかってもらうこと 4. 自分のストレス解消 5. パートナー（奥様や旦那様など）のストレス解消 6. 子ども**「●歳の●の子」**の学習や習い事 7. 子ども**「●歳の●の子」**の運動不足 8. 子ども**「●歳の●の子」**のストレス 9. 家庭の食事 10. 経済的なこと 11. マスクが足りないこと 12. 家庭内でもめ事が多くなったこと 13. その他（　　　　　　　　　　　　　　　　） 14. 困っていることは特にない |
| --- |

同居チェック：選択肢１～13と14を同時回答→アラート

［改ページ］

問7．あなたについて、次の質問にお答えください。これは、国の国民生活基礎調査でも実施されている心理的ストレスを評価する質問票です。最近の状況についてお答えください。（それぞれ１つずつ）

| 質問 | いつも感じている | たいてい感じている | ときどき感じている | 少しだけ感じている | まったく感じていない |
| --- | --- | --- | --- | --- | --- |
| 神経過敏に感じましたか | １ | ２ | ３ | ４ | ５ |
| 絶望的だと感じましたか | １ | ２ | ３ | ４ | ５ |
| そわそわ、落ち着かなく感じましたか | １ | ２ | ３ | ４ | ５ |
| 気分が沈み込んで何が起こっても気が晴れないように感じましたか | １ | ２ | ３ | ４ | ５ |
| 何をするのも骨折りだと感じましたか | １ | ２ | ３ | ４ | ５ |
| 自分は価値のない人間だと感じましたか | １ | ２ | ３ | ４ | ５ |

［改ページ］

問8．あなたのお子さん**「●歳の●の子」**は、手洗い、咳エチケットの感染予防はできていますか。
（１つだけ）

| 1. 完璧にできている 2. ほぼできている 3. まあまあできている 4. あまりできていない 5. できていない |
| --- |

［改ページ］

問9．あなたのお子さん**「●歳の●の子」**は、外で遊んでいますか。（１つだけ）

| 1. ほぼ毎日、外で遊んでいる 2. 週に3日～5日程度、外で遊んでいる 3. 週に１日か２日程度、外で遊んでいる 4. ほとんど外に出ない |
| --- |

［改ページ］

問10－１．あなたのお子さん**「●歳の●の子」の**、休園・休校時のLINEやゲーム、YouTubeなどの利用状況はどうですか。（１つだけ）

| 1. いつもの3倍以上の時間、利用している 2. いつもの２倍程度の時間、利用している 3. いつもと同程度の時間、利用している 4. いつもより利用している時間は少ない 5. 利用していない |
| --- |

［改ページ］

**【問１０－１で「１～４」と回答の方に】**

問10－２．あなたのお子さん**「●歳の●の子」の**、休園・休校時のLINEやゲーム、YouTubeなどの、１日の利用時間をお答えください。（数字で回答）

　　１日の利用時間は　　　　　　　　時間

　　（１～２４時間の回答制御）

［改ページ］

問11．次の質問は厚生労働省が紹介しているお子さんの子どもの心の健康に関連する質問です。保護者の方がご自身のお子さん**「●歳の●の子」**のことについてお答えください。

悩みやストレスが大きくなって、こころがダウンしそうなとき、様々なサインが現れます。特に、こころのＳＯＳは睡眠、食欲、体調、行動の4つの面に出てくることが多いでしょう。「今まではこんなことなかった」「どうも普段の様子と違う」など、いつもと違うことへの気づきが大切です。次のようなサインが続いているようなら、子どもから話を聞いてみましょう。そして、つらい症状が続いている場合は、専門家に相談してみることをお勧めします。

　あてはまることについて、いくつでも回答してください。（いくつでも）

睡眠：よく眠れること、十分な睡眠はこころの健康にとって大切です。

| 布団に入っても、なかなか寝つけないようだ。 | １ |
| --- | --- |
| 遅くまで夜更かししている。 | ２ |
| 朝、起きるのがつらそう、なかなか起きられない。 | ３ |
| 睡眠のリズムがくずれている。 | ４ |
| 眠れないと言う。 | ５ |
| 寝すぎる。 | ６ |

食欲：ストレスやこころの病から食欲に影響が及ぶことがあります。

| 食欲がない、食べる量が減った。 | ７ |
| --- | --- |
| 逆に食べすぎる。 | ８ |
| とくにパンやご飯、お菓子などの炭水化物を欲しがる。 | ９ |
| 急にやせた、あるいは太った。 | 10 |
| 体重をとても気にしている。 | 11 |

体調：こころの病気も、最初は体調に出てくることがよくあります。

| 体がだるそう。 | 12 |
| --- | --- |
| 疲れている。 | 13 |
| 元気がない。 | 14 |
| 顔色が悪い。 | 15 |
| 腹痛や頭痛、めまい、吐き気などを訴える。 | 16 |

行動：本人よりも周囲が気づきやすいのが行動面のサインです。

| 学校に行きたがらない。 | 17 |
| --- | --- |
| 家から出ないでひきこもりがちになった。 | 18 |
| 友達と遊ばなくなった。 | 19 |
| 身だしなみにかまわなくなった。 | 20 |
| 無口になった。 | 21 |
| 挨拶をしなくなった。 | 22 |
| 何度も同じ動作や行動をくりかえす。 | 23 |
| 気持ちが抑えられなくなり暴力をふるう。 | 24 |
| 何もしないで長い間ぼんやりしている。 | 25 |
| 表情が変わらず、感情面での反応が少なくなった。 | 26 |
| 話が支離滅裂になった、通じなくなった。 | 27 |
| 独り言を言うようになった。 | 28 |

| あてはまるものはない | 29 |
| --- | --- |

以上のようなサインがあるからといって、必ずしもこころの病気とは限りません。ただ、これまでなかったのに、このようなサインが見受けられるようになった場合や、長く続くような場合は、それはこころのＳＯＳなのかもしれません。

こころの病気は、多くの場合、早期に治療するほど回復も早くなるといわれています。ですから子どもの「いつもと違う」サインをキャッチしたら、早めに専門家に相談してみましょう。

［改ページ］

問12．新型コロナウイルス対策でお感じになっていることをご自由にお書きください

|  |
| --- |

［改ページ］

以上です。ご協力をありがとうございました。
