# Supplementary material for: Caregivers’ mental distress and child health during the COVID-19 outbreak in Japan
Source: PLoS One. 2020 Dec 10;15(12):e0243702. doi: 10.1371/journal.pone.0243702 (PMC7728265; doi:10.1371/journal.pone.0243702)
Supplement: S2 File — (DOCX) [file pone.0243702.s002.docx]

**[WEB survey (2020-04xxx)]**

| **Survey on child's health and life during the COVID-19 outbreak** |
| --- |

| **First of all, let us ask you about your gender and age.** |
| --- |

**[For all]**

SC1. Please tell us about your gender. (Only one)

| **1**woman  **2**men |
| --- |

[New Page]

SC2. Please tell us your age. (Only one)

| **1**Under 20 years old  **2**20-24 years old  **3**25-29 years old  **4**30-34 years old  **5**35-39 years old  **6**40-44 years old  **7**Over 45 years old |
| --- |

[New Page]

SC3. Do you have your own children who currently live with you? If so, please indicate the gender and age of your child. (All that apply)

| **1 No**children live together ( → Survey completed)  **2**Boys under 2 years old  **3**3-year-old boy (younger class in kindergarten / nursery in early April)  **4**4-year-old boy (year-round class for kindergarten and nursery school in early April)  **5**5 -year-old boy (senior class of kindergartens and nursery schools at the beginning of April)  **6**6- year-old boy (first grader in early April)  **7**7-year-old boy (2nd grade in early April)  **8**8-year-old boy (third grade elementary school at the beginning of April)  **9**9-year-old boy (fourth grade elementary school at the beginning of April)  **10**10-year-old boy (5th grade in early April)  **11**11-year-old boy (6th grade in early April)  **12**12-year-old boy (first grader in junior high school in early April)  **13**13-year-old boy (second grade in junior high school in early April)  **14**14-year-old boy (3rd year junior high school student in early April)  **15**Boy over 15 years old (high school students and above in early April)  **16**2-year-old girl  **17**3-year-old girl (younger class of kindergarten and nursery schools at the beginning of April)  **18**4-year-old girl (year-round class for kindergarten and nursery school in early April)  **19**5-year-old girl (older class in kindergarten / nursery in early April)  **20**6 -year-old woman of child (1 year of elementary school at the beginning of April)  **21**7 -year-old woman of child (second grade at the beginning of April)  **22**8-year-old girl (3rd grade in early April)  **23**9-year-old girl (4th grade in early April)  **24**10-year-old girl (5th grade in early April)  **25**11-year-old girl (6th grade in early April)  **26**12-year-old girl (first grader in junior high school in early April)  **27**13-year-old girl (second grade in junior high school in early April)  **28**14-year-old girl (3rd year middle school in early April)  **29**Girl over 15 years old (high school students and above in early April) |
| --- |

Cohabitation check: Option 1 and 2 to 29 simultaneous answer → Alerts

[New Page]

[For those who chose either 3-14 or 17-28 in SC3 (there is at least one boy / girl aged 3-14 years)]

SC4. Please read the following sentences carefully and answer if you can cooperate with this questionnaire.

**[Request for cooperation with a survey]**

The survey is conducted by Nippon Research Center commissioned by the Center for Birth Cohort Studies, University of Yamanashi.

The COVID-19 countermeasures placed great strain on mental health among children due to stress of staying at home, loneliness of not being able to meet friends, and fear of infection. There is urgent need to prevent child psychological issues while taking countermeasures against the COVID-19.

Therefore, the Center for Birth Cohort Studies, University of Yamanashi conducts this survey to grasp the current situation of children and parents during the countermeasures against the COVID-19 are in place, and to use the results for advising future countermeasures. The results of this survey will be analyzed and reported at academic conferences and journals.

　We appreciate your cooperation with this survey.

In this survey, we will ask you about the health condition of you and your child, but your answers will be anonymized and be used only for analyses at the Center for Birth Cohort Studies, University of Yamanashi. We will never use your name or answer for other purposes.

Can you cooperate with this survey? (Only one)

| **1**Yes, I can cooperate with this survey ( → Go to SC1 )  **2** No, I cannot cooperate with this survey ( → Survey completed) |
| --- |

[New Page]

Those who chose “1” in SC 4

To this survey (Q1)

| **Please answer each of the following questions.** |
| --- |

Question 1. Please indicate which prefecture you currently live. (Only one)

| **1**Hokkaido  **2**Aomori  **3**Iwate  **4**Miyagi  **5**Akita  **6**Yamagata  **7**Fukushima  **8**Ibaraki  **9**Tochigi  **10**Gunma  **11**Saitama  **12**Chiba  **13**Tokyo  **14**Kanagawa  **15**Niigata  **16**Toyama  **17**Ishikawa  **18**Fukui  **19**Yamanashi  **20**Nagano  **21**Gifu  **22**Shizuoka  **23**Aichi | **24**Mie  **25**Shiga  **26**Kyoto  **27**Osaka  **28**Hyogo  **29**Nara  **30**Wakayama  **31**Tottori  **32**Shimane  **33**Okayama  **34**Hiroshima  **35**Yamaguchi  **36**Tokushima  **37**Kagawa  **38**Ehime  **39**Kochi  **40**Fukuoka  **41**Saga  **42**Nagasaki  **43**Kumamoto  **44**Oita  **45**Miyazaki  **46**Kagoshima  **47**Okinawa |
| --- | --- |

[New Page]

Question 2. What is the size of the city you currently live in? (Only one)

| **1**more than half a million  **2**300,000 to less than 50 million people  **3**100,000 to less than 300,000  **4**50,000 people - less than 10 million people  **5**30,000 to less than 50,000 people  **6**10,000 to less than 30,000  **7**Less than 10,000 |
| --- |

[New Page]

| **The following questions ask about yourself and your ● -year-old ● (boy/girl) child living together.** |
| --- |

　　* " ● " indicates the answer of SC3 (if there are multiple answers, it is randomly chosen.)

The same applies to the following.

Question 4 -1. Please tell us about nursery or school your child attends.

　　　　　Are the nursery/school currently closed? (Only one)

| **1**Currently closed  **2**Currently not closed |
| --- |

[New Page]

**[For those who answered "1 Currently closed" in Q4-1]**

Question 4-2. Please tell us about nursery or school your child attends.

　　　　　Please select the one that applies to you. (All that apply)

| **1**Still going to nursery/school as an exception  **2** Using after-school care  **3**Online lessons available  **4**None of above |
| --- |

[New Page]

**[For those who answered "1 is currently closed / closed" in Q4-1]**

Question 4- 3. Please tell us when your child’s nursery/school closure started.

 　　　　　　　　　　　Month 　　　　　　　　　　　　Date

(Limit the answer within 2 to 5) (Limit the answer within 1 to 31)

  Question 4-4. Please tell us when the nursery/school closure will end.

 　　　　　　　　　　　Month 　　　　　　　　　　　　Date (planned)

(Limit the answer within 5 to 6) (Limit the answer within 1 to 31)

[New Page]

Q5. Please tell us the working status of you and your partner (wife, husband, etc.) .

Q5-1. What is your job? (Only one)

| **1**Office worker  **2**Civil servant  **3**Self-employed / freelance  **4**Agriculture, Forestry and Fisheries  **5**Part part-time job  **6**Full-time housework (home)  **7**Students  **8**Unemployed  **9**Others ( ) |
| --- |

[New Page]

Q5-2. How much time do you spend with your child during the daytime in weekdays (from Monday to Friday)? (Only one)

| **1**Stay together almost all day  **2** Stay together for about half a day  **3** Almost not together during the day |
| --- |

[New Page]

Q5-3. What is your partner’s job? (Only one)

| **1**Office worker  **2**Civil servant  **3**Self-employed / freelance  **4**Agriculture, Forestry and Fisheries  **5**Part part-time job  **6**Full-time housework (home)  **7**Students  **8**Unemployed  **9**Others ( ) |
| --- |

[New Page]

Q5-4. How much time do your partner spend with your child during the daytime in weekdays (from Monday to Friday)? (Only one)

| **1**Stay together almost all day  **2** Stay together for about half a day  **3** Almost not together during the day |
| --- |

[New Page]

Question 6. What is your concern? Please choose anything that applies. (All that apply)

| **1**My work / housework  **2**Partners (wife, husband, etc.) work / housework  **3**Have a child participate in a nursery/school  **4**Relieve your stress  **5** Relieve your partner’s stress (wife, husband, etc.)  **6**Education of your child  **7** Your child being lack of exercise  **8**Psychological stress of your child  **9**Home meals  **10**Household income and economic situation  **11**Difficulty in getting masks  **12**Increased troubles at home  **13**Others ( )  **14**I have no problems. |
| --- |

[New Page]

Question 7. Please answer the following questions about you. This is a questionnaire that evaluates psychological stress, which is also conducted in the Comprehensive Survey of Living Conditions. Please tell us about the recent situation. (Choose one for each)

| During the past 30 days, about how often did you feel … | All of the time | Most of the time | Some of the time | A little of the time | None of the time |
| --- | --- | --- | --- | --- | --- |
| 1. …nervous? | 1 | 2 | 3 | 4 | 5 |
| 1. …hopeless? | 1 | 2 | 3 | 4 | 5 |
| 1. …restless or fidgety? | 1 | 2 | 3 | 4 | 5 |
| 1. …so depressed that nothing could cheer you up? | 1 | 2 | 3 | 4 | 5 |
| e. …that everything was an effort? | 1 | 2 | 3 | 4 | 5 |
| f. …worthless? | 1 | 2 | 3 | 4 | 5 |

[New Page]

Question 8.  Is your child able to wash their hands and conduct coughing etiquette? (Only one)

| **1**Perfectly  **2**Almost  **3**OK  **4**Not very well  **5**No |
| --- |

[New Page]

Question 9. Does your child play outside? (Only one)

| **1**Play outside almost every day  **2**Play outside for 3 to 5 days per week  **3**Play outside for one or two days per week  **4**Hardly go out |
| --- |

[New Page]

Question 10-1. How much does your child spend watching/playing LINE/games/YouTube etc. during the nursery/school closure? (Only one)

| **1**More than 3 times as long as usual  **2**About twice as long as usual  **3**The same amount of time as usual  **4**Less time than usual  **5**None |
| --- |

[New Page]

**[For those who answered "1-4" in Q10-1]**

Question 10-2. Please tell us the time your child spends watching LINE, games, YouTube, etc. per day during the nursery/school closure. (Answer with numbers)

 　　hours per day

 　　(Limit the answer within 1 to 24 hours)

[New Page]

Question 11. The following questions are related to the mental health of your child, which is introduced by the Ministry of Health, Labor and Welfare. Please answer about your child **" ● year-old ●"**.

Various signs appear when your worries and stress grow, and your mind is about to go down. In particular, the SOS sign will often appear in four aspects: sleep, appetite, physical and psychological condition, and behavior. It is important to be aware of signs that are different from usual, such as "this has never happened before" and "it looks different than usual". If you see the following signs, talk to your child. And if you continue to see the symptoms, it might be worth consulting a specialist.

Please choose all that apply.

Sleep: Good sleep and good sleep are important for your mental health.

| Even if he/she goes to bed, it seems that he/she can't sleep easily. | 1 |
| --- | --- |
| He/She stays up late until late. | 2 |
| It's hard to get up in the morning. | 3 |
| The rhythm of sleep is broken. | 4 |
| He/She says “I can't sleep”. | 5 |
| He/She sleeps too much. | 6 |

Appetite: Stress and mental illness can affect appetite.

| He/She has no appetite and eats less. | 7 |
| --- | --- |
| He/She eats too much. | 8 |
| He/She wants carbohydrates such as bread, rice, and sweets. | 9 |
| He/She lost or gained weight dramatically. | Ten |
| He/She is very concerned about his/her weight. | 11 |

Physical condition: Mental illnesses often appear in physical condition at first.

| He/She feels uncomfortable. | 12 |
| --- | --- |
| He/She looks tired. | 13 |
| He/She is not feeling well. | 14 |
| He/She looks sick. | 15 |
| He/She complains of abdominal pain, headache, dizziness and nausea. | 16 |

Behavior: Behavioral signs are easier to notice than the person in question.

| He/She doesn't want to go to school. | 17 |
| --- | --- |
| He/She shuts his/herself in at home. | 18 |
| He/She stopped playing with my friends. | 19 |
| He/She doesn't care about his/her appearance. | 20 |
| He/She became silent. | 21 |
| He/She stopped greeting. | 22 |
| He/She repeats the same actions over and over again. | 23 |
| He/She can't control his/her feelings and uses violence. | 24 |
| He/She has been absent-minded for a long time without doing anything. | 25 |
| His/Her facial expression did not change, and his/her emotional reaction decreased. | 26 |
| His/Her story became incoherent and incomprehensible. | 27 |
| He/She started to talk to myself. | 28 |

| None of the above | 29 |
| --- | --- |

The above signs do not necessarily mean that your child has a mental illness. However, if you can see such a sign, or if the sign lasts for a long time, it may be the SOS from your child.

The earlier intervention will lead to the faster recovery of mental illness. So, if you find a child's "unusual" sign, consult a specialist as soon as possible.

[New Page]

Question 12. Feel free to write what you feel about the COVID-19 countermeasures.

|  |
| --- |

[New Page]

 This is the end of the survey. We appreciate your cooperation.
